# Supplementary figures and images for: The giant mimivirus 1.2 Mb genome is elegantly organized into a 30-nm diameter helical protein shield
Source: eLife. 2022 Jul 28;11:e77607. doi: 10.7554/eLife.77607 (PMC9512402; doi:10.7554/eLife.77607)

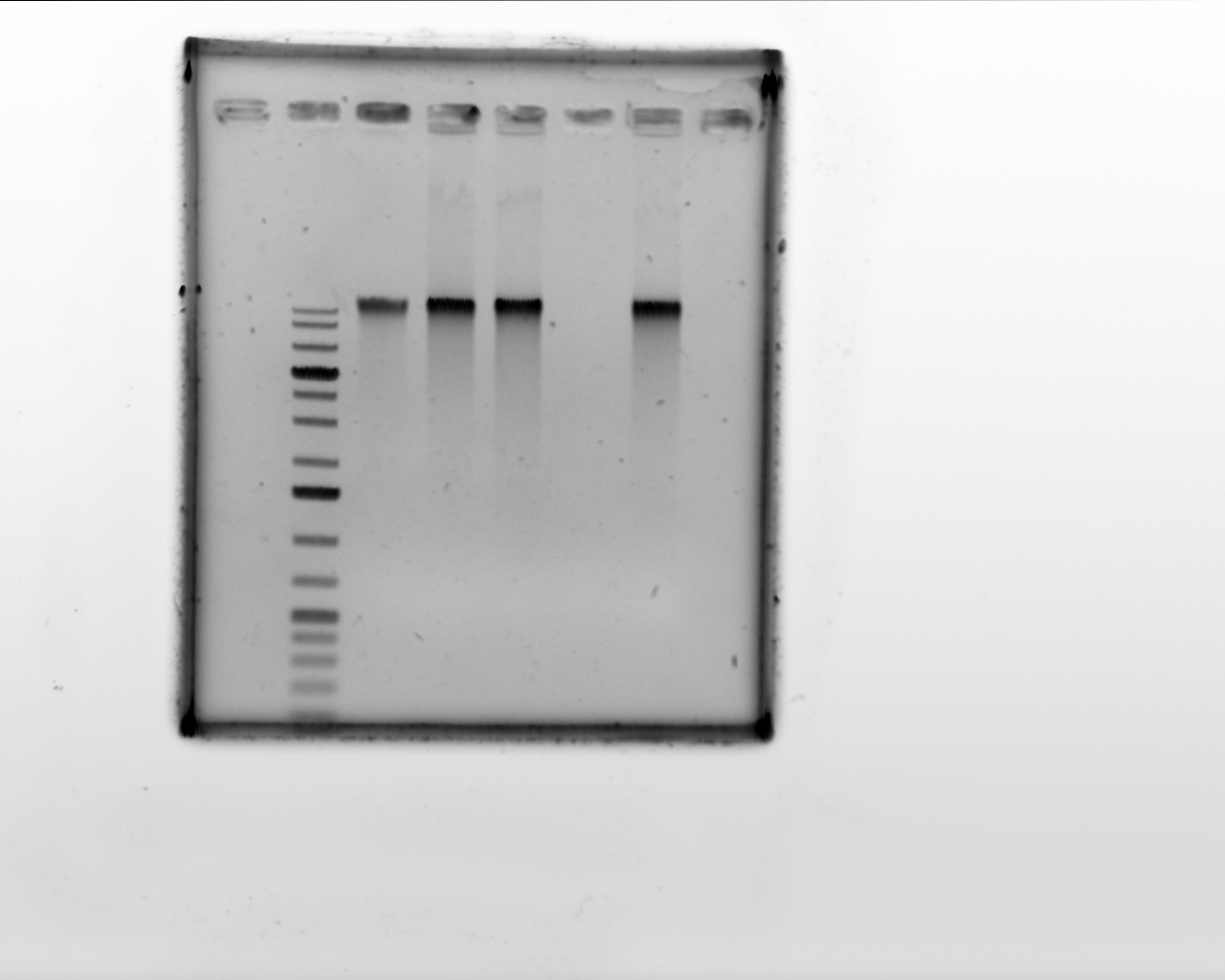

Supplement: Figure 1—figure supplement 3—source data 2. [file elife-77607-fig1-figsupp3-data2.zip › Figure 1-figure supplement 2-source data 1.Tif]

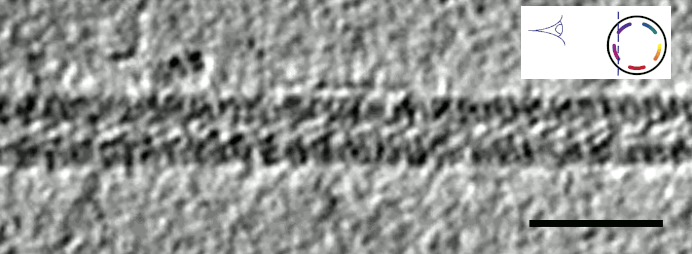

Supplement: Supplementary file 9 [file elife-77607-fig3-video2.gif]
