# Supplementary material for: The giant mimivirus 1.2 Mb genome is elegantly organized into a 30-nm diameter helical protein shield
Source: eLife. 2022 Jul 28;11:e77607. doi: 10.7554/eLife.77607 (PMC9512402; doi:10.7554/eLife.77607)
Supplement: Supplementary file 1. [file elife-77607-supp1.docx]

**Supplementary file 1:** **Mimivirus Reunion genomic fibers data statistics** (*33*)

| **Mimivirus Reunion** | **5-start fiber** | | | **6-start fiber** |
| --- | --- | --- | --- | --- |
|  | **Asymmetric unit**  **(Cl1a)** | **Compacted helix**  **(Cl1a)** | **Relaxed helix**  **(Cl3a)** | **(Cl2)** |
| **Dimensions and symmetries** | | | | |
| Protein shell width  (external, internal) (Å) | - | 286.2, 117.7 | 324.5, 168.5 | 312.0, 151.4 |
| Shell thickness (Å) | - | 84.2 | 78 | 80.3 |
| DNA ring diameter  (external, internal) (Å) | - | 132.0, 93.15 | - | 163.6, 124.4 |
| Spacing Shell-DNA | - | 5.5 | - | 4.8 |
| **3D Refinement** (Fig. S6-8) | | | | |
| Symmetry imposed | C1 | Helical | Helical, D5 | Helical, C3 |
| Helical parameters  (rise, twist) | - | 7.93 Å, -221.075° | 31.11 Å, -23.975° | 20.47 Å, 49.43° |
| Initial particle images (#) | 418,041 | 418,041 | 107,004 | 162,379 |
| Final particle images (#) | 95,722 | 121.429 | 11,958 | 8,479 |
| Initial model used | Low-pass filtered reconstruction from subtracted segments | Featureless cylinder | | |
| Model resolution  (Å) (masked) | 3.3 | 3.7 | 3.7 | 4.0 |
| FSC threshold | 0.5 | 0.5 | 0.5 | 0.5 |
| **Asymmetric unit model statistics** | | | | |
| *Model composition* | | | | |
| Non-hydrogen atoms | 9,996 | 10,072 | 4,998 | 10,124 |
| Protein residues | 1,290 | 1,300 | 645 | 1,298 |
| *R.m.s. deviations* | | | |  |
| Bond lengths (Å) | 0.008 (0) | 0.007 (0) | 0.006 (0) | 0.006 (0) |
| Bond angles (°) | 1.134 (0) | 1.194 (10) | 0.846 (1) | 0.802 (0) |
| *Validation* | | | | |
| MolProbity score | 2.18 | 2.73 | 2.36 | 2.16 |
| Clashscore | 8.20 | 16.18 | 17.12 | 14.27 |
| Rotamers outliers (%) | 2.05 | 4.61 | 1.49 | 0.00 |
| *Ramachandran plot* | | | |  |
| Favoured (%) | 91.60 | 91.13 | 91.76 | 91.58 |
| Allowed (%) | 8.40 | 8.87 | 8.24 | 8.42 |
| Disallowed (%) | 0.00 | 0.00 | 0.00 | 0.00 |
