# Supplementary material for: The giant mimivirus 1.2 Mb genome is elegantly organized into a 30-nm diameter helical protein shield
Source: eLife. 2022 Jul 28;11:e77607. doi: 10.7554/eLife.77607 (PMC9512402; doi:10.7554/eLife.77607)
Supplement: Supplementary file 2. — (ND: not detected). RNA polymerase subunits are marked in red. [file elife-77607-supp2.docx]

**Supplementary file 2: Mass spectrometry-based proteomic analysis** of A] three independent preparations of Mimivirus genomic fiber and B] one sample of purified Mimivirus virions. (ND: Not detected). RNA polymerase subunits are marked in red.

**A**

| **accession** | **protein name** | **molecular weight** | **peptides** | **Coverage**  **%** | **iBAQ R1** | **iBAQ R2** | **iBAQ R3** | **Copies/fiber** | **Standard deviation** |
| --- | --- | --- | --- | --- | --- | --- | --- | --- | --- |
| qu_946 | GMC oxidoreductase | 78892 | 59 | 65.88 | 25605966 | 59667499 | 95078727 | 95378 |  |
| qu_143 | GMC-type oxidoreductase | 76945 | 49 | 56.27 | 5056660 | 27839892 | 10688103 |  |  |
| qu_734 | hypothetical protein | 23302 | 5 | 29.28 | 2567070 | 6045037 | 7467041 | 7102 | 768 |
| qu_313 | kinesin-like protein | 339986 | 135 | 37.95 | 29583 | 473 | 3596453 | 1112 | 1846 |
| qu_446 | Major capsid protein D13L | 66124 | 21 | 38.21 | 246585 | 836883 | 690005 | 767 | 145 |
| qu_431 | Core protein | 75338 | 19 | 21.79 | 142618 | 31426 | 1026459 | 468 | 446 |
| qu_572 | VVI8 helicase | 79516 | 17 | 24.03 | 221920 | ND | 106288 | 393 | 420 |
| qu_772 | hypothetical protein | 24047 | 8 | 34.4 | 108330 | 670995 | 86097 | 382 | 329 |
| qu_384 | Thioredoxin | 39459 | 9 | 14.74 | 175615 | 238801 | 302348 | 360 | 162 |
| qu_738 | hypothetical protein | 39948 | 6 | 15.54 | 150035 | 93504 | 379485 | 304 | 185 |
| qu_196 | putative amine oxidase | 56642 | 11 | 26.52 | 36721 | 500469 | 23509 | 227 | 280 |
| qu_76 | collagen-like protein 1 | 91015 | 6 | 5.25 | 37893 | 478053 | 38102 | 224 | 260 |
| **qu_245** | RNA polymerase subunit 5 | 23541 | 7 | 31.22 | 75069 | 45112 | 307708 | 187 | 121 |
| qu_623 | hypothetical protein | 29447 | 3 | 8.49 | 100753 | 178920 | 19727 | 175 | 149 |
| qu_351 | hypothetical protein | 30607 | 1 | 2.64 | 53353 | 180588 | 177860 | 174 | 20 |
| qu_231 | Topoisomerase I | 96906 | 11 | 15.69 | 100666 | ND | 39460 | 174 | 196 |
| qu_685 | hypothetical protein | 19573 | 1 | 7.57 | 70350 | 46933 | ND | 135 | 119 |
| qu_366 | Regulator of chromosome condensation | 107437 | 11 | 14.33 | 57761 | 89643 | 109833 | 125 | 47 |
| qu_409 | hypothetical protein | 30040 | 3 | 11.32 | 26557 | 142452 | 28594 | 88 | 65 |
| qu_368 | hypothetical protein | 56316 | 4 | 8.04 | 30458 | 98708 | 64453 | 87 | 26 |
| **qu_493** | DNA directed RNA polymerase subunit (Rpb3/11) | 41630 | 8 | 24.09 | 30508 | 16795 | 162795 | 87 | 65 |
| qu_268 | hypothetical protein | 38970 | 3 | 8.99 | 31211 | 36923 | 130386 | 85 | 40 |
| **qu_261-259-257-255** | DNA directed RNA polymerase subunit 2 | 135691 | 13 | 11.57 | 28814 | 21461 | 113619 | 72 | 42 |
| qu_752 | orphan | 34452 | 4 | 9.27 | 23162 | 2162 | 146936 | 69 | 65 |
| qu_880 | hypothetical protein | 49215 | 5 | 9.93 | 21357 | 93652 | 39853 | 68 | 33 |
| qu_464 | hypothetical protein | 139105 | 12 | 9.37 | 39403 | 16346 | 68323 | 67 | 53 |
| qu_753 | hypothetical protein | 49938 | 6 | 12.86 | 14018 | ND | 93259 | 64 | 29 |
| **qu_379** | DNA-directed RNA polymerase II subunit N | 8860 | 2 | 35.62 | 29494 | 14124 | 93245 | 64 | 42 |
| qu_544 | hypothetical protein | 10721 | 2 | 11.83 | 26508 | 56949 | 47381 | 62 | 20 |
| qu_424 | hypothetical protein | 87443 | 12 | 12.07 | 12589 | 610 | 158643 | 61 | 74 |
| **qu_530-532** | DNA directed RNA polymerase II subunit 1 | 119306 | 8 | 6.23 | 18279 | 10404 | 86427 | 49 | 34 |
| **qu_219** | DNA-dependent RNA polymerase subunit Rpb9 | 22289 | 2 | 19.79 | 11155 | ND | 51304 | 40 | 8 |
| qu_773 | hypothetical protein | 26538 | 2 | 8.48 | 15353 | 32905 | 19722 | 34 | 15 |
| qu_495 | hypothetical protein | 199755 | 11 | 6.59 | 10508 | 11663 | 53398 | 31 | 18 |
| qu_629 | Thiol oxidoreductase E10R | 34053 | 2 | 7.19 | ND | 39151 | 11930 | 27 | 23 |
| qu_582 | hypothetical protein | 16640 | 1 | 12.57 | 12335 | 12718 | 18499 | 23 | 13 |
| qu_508 | ankyrin repeat-containing protein | 219141 | 5 | 2 | 2519 | ND | 35112 | 20 | 17 |
| qu_947 | endonuclease/exonuclease/phosphatase | 79875 | 1 | 1.72 | 3834 | 24132 | 8897 | 15 | 10 |
| qu_710 | Collagen triple helix repeat containing protein | 191259 | 1 | 0.58 | 3963 | 25823 | 1854 | 14 | 13 |
| qu_371 | transcription termination factor | 95426 | 4 | 6 | 9662 | 2229 | 8306 | 13 | 15 |
| qu_686 | NHL repeat-containing protein | 43392 | 1 | 2 | 3438 | 15317 | 11025 | 12 | 4 |
| qu_420 | hypothetical protein | 39401 | 1 | 3.12 | 6273 | 5066 | 12842 | 12 | 7 |
| qu_347 | Early transcription factor large subunit | 245225 | 3 | 1.89 | 3038 | ND | 7791 | 8 | 2 |
| qu_585 | hypothetical protein | 56754 | 1 | 2.51 | ND | 7107 | 3281 | 5 | 3 |
| qu_546 | hypothetical protein | 86695 | 1 | 2.61 | ND | 2195 | 3619 | 3 | 1 |
| qu_709 | collagen-like protein 6 | 141539 | 1 | 1.21 | ND | 2012 | 1539 | 2 | 1 |

**B**

| **accession** | **protein name** | **molecular weight** | **peptides** | **Coverage %** | **iBAQ** |
| --- | --- | --- | --- | --- | --- |
| qu_446 | Major capsid protein D13L | 66124 | 69 | 94.84 | 210053824 |
| qu_143 | GMC-type oxidoreductase | 76945 | 78 | 93.73 | 186790085 |
| qu_685 | hypothetical protein | 19573 | 6 | 32.43 | 174911824 |
| qu_544 | hypothetical protein | 10721 | 6 | 43.01 | 120277696 |
| qu_582 | hypothetical protein | 16640 | 5 | 40.72 | 116093496 |
| qu_464 | hypothetical protein | 139105 | 125 | 67.04 | 107226726 |
| qu_431 | Core protein | 75338 | 91 | 80.64 | 101971100 |
| qu_773 | hypothetical protein | 26538 | 23 | 78.12 | 99393566 |
| qu_669 | Orphan | 5784 | 2 | 98.33 | 92508352 |
| qu_384 | Thioredoxin | 39459 | 27 | 53.47 | 66455090 |
| qu_772 | hypothetical protein | 24047 | 22 | 86.24 | 53127153 |
| qu_482 | hypothetical protein | 21099 | 14 | 64.36 | 45300565 |
| qu_366 | Regulator of chromosome condensation | 107437 | 41 | 47.15 | 43178787 |
| qu_946 | GMC oxidoreductase | 78892 | 50 | 77.67 | 41561796 |
| qu_589 | hypothetical protein | 17955 | 10 | 81.88 | 32465195 |
| qu_600 | hypothetical protein | 37211 | 38 | 70 | 28534896 |
| qu_736 | hypothetical protein | 40921 | 26 | 77.54 | 28136889 |
| qu_657 | hypothetical protein | 28864 | 2 | 7.43 | 27887883 |
| qu_686 | NHL repeat-containing protein | 43392 | 20 | 75.75 | 27407813 |
| qu_738 | hypothetical protein | 39948 | 9 | 18.08 | 25518798 |
| qu_734 | hypothetical protein | 23302 | 8 | 31.53 | 25487740 |
| qu_409 | hypothetical protein | 30040 | 18 | 82.64 | 25169756 |
| qu_757 | hypothetical protein | 21968 | 11 | 72.16 | 24470704 |
| qu_480 | hypothetical protein | 21376 | 12 | 70.9 | 23375851 |
| qu_268 | hypothetical protein | 38970 | 34 | 85.22 | 20235521 |
| qu_465 | thioredoxin domain-containing protein | 19438 | 11 | 43.29 | 19680372 |
| qu_368 | hypothetical protein | 56316 | 38 | 67.22 | 15678817 |
| qu_585 | hypothetical protein | 56754 | 26 | 56.56 | 15502792 |
| qu_351 | hypothetical protein | 30607 | 16 | 66.42 | 13371044 |
| qu_684 | hypothetical protein | 30286 | 6 | 59.34 | 13325675 |
| qu_345 | hypothetical protein | 39318 | 17 | 33.62 | 13075398 |
| qu_511 | putative PAN domain-containing protein | 24425 | 4 | 22.87 | 12445425 |
| qu_515 | hypothetical protein | 25475 | 16 | 57.01 | 12413518 |
| qu_514 | hypothetical protein | 63202 | 25 | 27.91 | 11786549 |
| qu_420 | hypothetical protein | 39401 | 12 | 16.48 | 11185321 |
| qu_423 | hypothetical protein | 91000 | 31 | 40.42 | 11166382 |
| qu_775 | hypothetical protein | 32226 | 12 | 21.88 | 10813839 |
| qu_307 | Ricin-type lectin protein | 22911 | 3 | 30.05 | 10519539 |
| qu_824 | putative PAN domain-containing protein | 22435 | 4 | 29 | 10383817 |
| qu_543 | hypothetical protein | 18141 | 3 | 22.62 | 9999289 |
| qu_828 | hypothetical protein | 28776 | 13 | 68.09 | 9907607 |
| qu_752 | Orphan | 34452 | 8 | 20.77 | 9661887 |
| qu_367 | hypothetical protein | 21736 | 5 | 51.28 | 9189856 |
| qu_644 | hypothetical protein | 37344 | 21 | 67.77 | 8623543 |
| qu_623 | hypothetical protein | 29447 | 13 | 76.83 | 8397789 |
| qu_557 | putative triacylglycerol lipase | 38826 | 36 | 80.99 | 8251842 |
| qu_529 | hypothetical protein glt_00638 | 9907 | 3 | 32.53 | 7975426 |
| qu_500 | Orphan | 11233 | 2 | 24.49 | 7372987 |
| qu_509 | hypothetical protein | 19344 | 9 | 44.12 | 7320892 |
| qu_245 | RNA polymerase subunit 5 | 23541 | 18 | 77.56 | 6774205 |
| qu_767 | hypothetical protein | 37663 | 22 | 52.77 | 6545302 |
| qu_683 | low complexity hypothetical protein | 47185 | 6 | 22.28 | 6521862 |
| qu_692 | hypothetical protein | 26417 | 16 | 96.48 | 5818265 |
| qu_616 | uncharacterized N-acetyltransferase | 46994 | 21 | 53.07 | 5610844 |
| qu_617 | hypothetical protein | 11815 | 4 | 43.93 | 5271258 |
| qu_629 | Thiol oxidoreductase E10R | 34053 | 16 | 56.85 | 5134527 |
| qu_169 | hypothetical protein | 51058 | 27 | 65.59 | 4990008 |
| **qu_530-532** | DNA directed RNA polymerase II subunit 1 | 119306 | 67 | 54.07 | 4778589 |
| qu_646 | uncharacterized cupin RmlC-type domain protein | 18499 | 7 | 47.5 | 4728743 |
| qu_280 | uncharacterized WD repeat-containing protein | 153562 | 25 | 25.85 | 4670418 |
| qu_64 | hypothetical protein | 21868 | 5 | 20 | 4542134 |
| qu_70 | uncharacterized virion-associated membrane protein | 39117 | 11 | 52.68 | 4370076 |
| qu_880 | hypothetical protein | 49215 | 20 | 51.27 | 4315781 |
| qu_371 | Transcription termination factor | 95426 | 48 | 62.67 | 4179959 |
| **qu_261-259-257-255** | DNA directed RNA polymerase subunit 2 | 135691 | 59 | 53.9 | 4071877 |
| qu_454 | hypothetical protein | 17244 | 4 | 64.78 | 4031133 |
| qu_748 | hypothetical protein | 22233 | 7 | 57.14 | 3838958 |
| qu_578 | hypothetical protein | 23850 | 5 | 17.37 | 3754039 |
| qu_328 | hypothetical protein | 8110 | 2 | 40.85 | 3683040 |
| qu_681 | hypothetical protein | 34943 | 3 | 9.52 | 3595732 |
| qu_495 | hypothetical protein | 199755 | 90 | 53.82 | 3589218 |
| qu_737 | hypothetical protein | 32336 | 8 | 39.27 | 3565424 |
| **qu_493** | DNA directed RNA polymerase subunit | 41630 | 18 | 44.26 | 3551856 |
| qu_55 | hypothetical protein | 8296 | 3 | 39.19 | 3403650 |
| qu_546 | hypothetical protein | 86695 | 50 | 68.72 | 3333680 |
| qu_625 | putative prolyl 4-hydroxylase | 27901 | 9 | 45.87 | 3078983 |
| qu_267 | hypothetical protein | 28538 | 3 | 14.55 | 3058533 |
| qu_344 | uncharacterized myristoylated membrane protein | 29395 | 4 | 39.93 | 2959488 |
| qu_769 | putative chemotaxis protein CheD | 31169 | 10 | 41.85 | 2945006 |
| qu_196 | putative amine oxidase | 56642 | 19 | 43.81 | 2924113 |
| qu_313 | kinesin-like protein | 339986 | 139 | 53.33 | 2911617 |
| **qu_220** | DNA-directed RNA polymerase subunit 6 | 46141 | 11 | 30.75 | 2885042 |
| qu_525 | probable zinc-type alcohol dehydrogenase-like protein | 47354 | 14 | 35.78 | 2756323 |
| qu_424 | hypothetical protein | 87443 | 37 | 54.11 | 2688682 |
| qu_205 | probable glutaredoxin | 12084 | 7 | 69.81 | 2630248 |
| qu_519 | hypothetical protein | 20432 | 5 | 32.96 | 2620066 |
| qu_404 | probable mRNA-capping enzyme | 136590 | 56 | 54.96 | 2518068 |
| qu_361 | poxvirus polyA polymerase catalytic subunit-like protein | 67815 | 32 | 47.76 | 2471347 |
| **qu_398** | DNA-directed RNA polymerase subunit | 22532 | 10 | 61.31 | 2407246 |
| qu_204 | DNA topoisomerase 1b | 39906 | 18 | 50 | 2271146 |
| qu_405 | divergent methyltransferase | 40370 | 12 | 36.21 | 2145355 |
| qu_618 | hypothetical protein | 11580 | 3 | 45.63 | 2106599 |
| qu_419 | hypothetical protein MIMI_R398 | 41246 | 11 | 37.85 | 1998969 |
| qu_753 | hypothetical protein | 49938 | 20 | 50.71 | 1926494 |
| qu_349 | hypothetical protein | 9695 | 2 | 30.59 | 1882708 |
| qu_924 | hypothetical protein | 39477 | 17 | 55 | 1858931 |
| qu_170 | hypothetical protein | 50940 | 22 | 60.05 | 1822949 |
| qu_325 | putative protein phosphatase 2C | 33565 | 8 | 31.16 | 1764492 |
| qu_477 | hypothetical protein | 142243 | 46 | 43.99 | 1761589 |
| qu_724 | putative serine/threonine-protein kinase | 80157 | 24 | 42.8 | 1721199 |
| qu_508 | ankyrin repeat-containing protein | 219141 | 76 | 52.37 | 1649278 |
| qu_697 | hypothetical protein | 62887 | 26 | 54.9 | 1622542 |
| qu_399 | putative NTPase | 134005 | 47 | 47.17 | 1596235 |
| qu_338 | probable DNA polymerase family X | 40598 | 21 | 53.11 | 1530525 |
| qu_329 | hypothetical protein | 10979 | 3 | 45.26 | 1520464 |
| qu_486 | hypothetical protein | 32747 | 7 | 27.76 | 1436853 |
| qu_246 | hypothetical protein | 17750 | 6 | 62.16 | 1391920 |
| qu_390 | probable FAD-linked sulfhydryl oxidase | 16981 | 7 | 62.24 | 1379940 |
| qu_747 | Serpin | 46879 | 17 | 50.87 | 1379737 |
| qu_639 | structural PPIase-like protein | 26569 | 8 | 31.62 | 1216625 |
| **qu_379** | DNA-directed RNA polymerase II subunit N | 8860 | 2 | 54.79 | 1197770 |
| qu_438 | hypothetical protein | 56175 | 16 | 34.42 | 1173947 |
| qu_643 | hypothetical protein | 38822 | 4 | 12.95 | 1169692 |
| qu_547 | hypothetical protein | 76930 | 23 | 53.69 | 1163885 |
| qu_932 | putative lipocalin | 19105 | 3 | 14.79 | 1096271 |
| qu_347 | Early transcription factor large subunit | 245225 | 65 | 37.11 | 1085524 |
| qu_656 | putative tyrosine-protein phosphatase | 24840 | 5 | 24.06 | 991502 |
| qu_770 | putative bifunctional metalloprotease/ubiquitin-protein ligase | 100069 | 20 | 34.86 | 960569 |
| qu_572 | VVI8 helicase | 79516 | 21 | 42.01 | 920005 |
| qu_76 | collagen-like protein 1 | 91015 | 3 | 3.61 | 914169 |
| qu_682 | phosphatidylethanolamine-binding protein-like protein | 16882 | 9 | 83.92 | 864819 |
| qu_596 | putative ATP-dependent RNA helicase | 66972 | 19 | 46.42 | 819446 |
| qu_376 | putative thiol protease | 34896 | 13 | 60.93 | 766743 |
| qu_563 | hypothetical protein | 59781 | 16 | 34.06 | 760199 |
| qu_378 | hypothetical protein | 57328 | 7 | 20.32 | 734789 |
| qu_327 | Uncharacterized protein L309 | 36716 | 3 | 11.53 | 720399 |
| qu_231 | Topoisomerase I | 96906 | 21 | 29.86 | 708206 |
| **qu_219** | DNA-dependent RNA polymerase subunit Rpb9 | 22289 | 2 | 19.79 | 683379 |
| qu_430 | hypothetical protein | 20862 | 4 | 38.38 | 662120 |
| qu_383 | hypothetical protein | 30954 | 4 | 29.01 | 649615 |
| qu_346 | uncharacterized metallopeptidase WLM domain protein | 22781 | 6 | 37.56 | 599956 |
| qu_23 | hypothetical protein | 21233 | 3 | 19.13 | 592113 |
| qu_558 | XRN 5'-3' exonuclease | 93111 | 24 | 35.92 | 573028 |
| qu_421 | F10-like kinase | 54385 | 11 | 27.84 | 550330 |
| qu_312 | peptidase C19 subfamily protein | 51173 | 9 | 28.76 | 538715 |
| qu_496 | hypothetical protein | 30933 | 5 | 35.91 | 521884 |
| qu_324 | hypothetical protein | 19980 | 3 | 28.4 | 510212 |
| qu_412 | hypothetical protein | 89610 | 12 | 23.59 | 486614 |
| qu_335 | probable formamidopyrimidine-DNA glycosylase | 33463 | 13 | 60.63 | 456129 |
| qu_900 | hypothetical protein | 11477 | 3 | 31 | 455575 |
| qu_382 | hypothetical protein | 21290 | 7 | 44.63 | 455329 |
| qu_638 | hypothetical protein | 11215 | 1 | 12.63 | 448838 |
| qu_320 | putative serine/threonine-protein kinase | 47356 | 7 | 26.7 | 387208 |
| qu_678 | hypothetical protein | 59156 | 5 | 12.36 | 370254 |
| qu_322 | NAD-dependent DNA ligase | 72056 | 12 | 30.19 | 370074 |
| qu_373 | hypothetical protein | 33827 | 8 | 42.81 | 324966 |
| qu_584 | hypothetical protein | 35586 | 7 | 21.75 | 313721 |
| qu_571 | hypothetical protein | 32361 | 9 | 24.73 | 296379 |
| qu_411 | hypothetical protein | 127231 | 13 | 16.9 | 270613 |
| qu_221 | hypothetical protein | 45001 | 7 | 18.49 | 243762 |
| qu_762 | hypothetical protein | 13377 | 2 | 21.74 | 237330 |
| qu_729 | Uncharacterized protein L684 | 56486 | 7 | 20.41 | 226886 |
| qu_663 | hypothetical protein | 36625 | 5 | 20.19 | 206880 |
| qu_568 | putative ATP-dependent RNA helicase | 51600 | 7 | 24.49 | 190886 |
| qu_750 | hypothetical protein | 16574 | 2 | 15.56 | 169202 |
| qu_768 | endonuclease VIII-like protein | 37647 | 3 | 10.09 | 156676 |
| qu_733 | endonuclease for the repair of UV-irradiated DNA | 39578 | 5 | 23.01 | 154909 |
| qu_513 | hypothetical protein | 10593 | 2 | 45.16 | 153955 |
| qu_434 | hypothetical protein | 42079 | 5 | 20.56 | 144052 |
| qu_350 | hypothetical protein | 20898 | 3 | 28.81 | 142240 |
| qu_440 | uncharacterized short-chain type dehydrogenase/reductase | 37903 | 3 | 13.16 | 132212 |
| qu_576 | Chain A2C A Megaviridae Orfan Gene Encodes A New Nucleotidyl Transferase 4AMQ_A | 45873 | 6 | 18.6 | 111651 |
| qu_227 | hypothetical protein | 87553 | 8 | 16.8 | 99165 |
| qu_388 | putative ATP-dependent RNA helicase | 208612 | 17 | 15.01 | 89684 |
| qu_394 | hypothetical protein | 29429 | 2 | 9.6 | 89379 |
| qu_484 | hypothetical protein | 24362 | 1 | 3.83 | 81052 |
| qu_668 | Bulb-type mannose-specific lectin | 21574 | 1 | 14.07 | 78249 |
| qu_393 | putative ankyrin repeat protein | 90149 | 3 | 3.92 | 76679 |
| qu_909 | hypothetical protein | 36665 | 3 | 13.02 | 68006 |
| qu_475 | hypothetical protein | 52379 | 2 | 13.12 | 63506 |
| qu_466 | putative ADP-ribosyl glycohydrolase | 58740 | 3 | 9.75 | 58759 |
| qu_620 | hypothetical protein | 44621 | 2 | 6.55 | 56022 |
| qu_395 | uncharacterized glycosyltransferase | 29888 | 2 | 13.94 | 52627 |
| qu_315 | putative endonuclease 4 | 32949 | 2 | 14.58 | 51762 |
| qu_536 | hypothetical protein | 36584 | 1 | 4.06 | 45593 |
| qu_297 | hypothetical protein | 14835 | 2 | 11.11 | 40398 |
| qu_291 | hypothetical protein | 23506 | 1 | 5.19 | 39136 |
| qu_243 | putative Zn-dependent peptidase | 50666 | 2 | 4.09 | 37619 |
| qu_710 | Collagen triple helix repeat containing protein | 191259 | 2 | 2.25 | 25872 |
| qu_709 | collagen-like protein 6 | 141539 | 2 | 3.35 | 22882 |
| qu_225 | RAS family GTPase | 23738 | 1 | 5.31 | 20950 |
| qu_248 | Collagen triple helix repeat containing protein | 44720 | 1 | 2.72 | 20679 |
| qu_492 | putative bifunctional polynucleotide phosphatase/kinase | 49171 | 2 | 8.08 | 15756 |
| qu_963 | putative Fe2OG oxygenase family oxidoreductase | 25167 | 1 | 6.19 | 15033 |
| qu_418 | hypothetical protein | 64964 | 1 | 3.34 | 14304 |
| qu_461 | Capsid protein | 69341 | 2 | 4.57 | 11635 |
| qu_665 | hypothetical protein | 25427 | 1 | 7.49 | ND |
| qu_207 | collagen triple helix repeat containing protein | 131064 | 1 | 1.44 | ND |
