# Supplementary material for: The giant mimivirus 1.2 Mb genome is elegantly organized into a 30-nm diameter helical protein shield
Source: eLife. 2022 Jul 28;11:e77607. doi: 10.7554/eLife.77607 (PMC9512402; doi:10.7554/eLife.77607)
Supplement: Supplementary file 3. — Conserved or divergent amino acids are color coded in green or in red, respectively. [file elife-77607-supp3.docx]

**Supplementary file 3: Contacting residues for each GMC-oxidoreductases in the different maps**

Conserved or divergent amino-acids are color coded in green or in red, respectively.

| **Cl1a** | | **Cl2** | | **Cl3a** | |
| --- | --- | --- | --- | --- | --- |
| **qu946** | **qu143** | **qu946** | **qu143** | **qu946** | **qu143** |
| ***Intra-start (A-C and B-D)*** | | | | | |
| N96-N96 | N94-N94 | N96-N96 | N94-N94 | N96-N96 | N94-N94 |
| N114-D276 | N112-D274 | N114-D276 | N112-D274 | N114-D276 | N112-D274 |
| S275-N114 | S275-N114 | S275-K493 | S273-K493 | S275-N114 | S275-N114 |
| T700-S371 | N700-S369 |  | N700-S369 |  | N702-S369 |
| S275-D366 | D273-D364 | S275-D366 | D273-D364 |  |  |
| D366-S275 | D364-S273 |  |  | D366-S275 | D364-S273 |
| D276-E115 | D276-E115 |  |  |  |  |
| 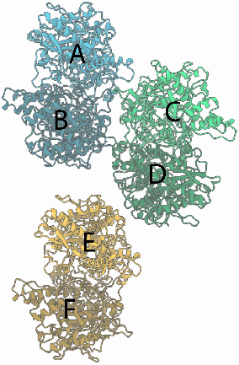 | |  |  |  | N700-T366 |
|  |  |  |  |  | N702-S370 |
|  |  | S371-T700 | S369-N700 |  |  |
|  |  |  | E294-E294 |  |  |
|  |  |  | N295-N295 |  |  |
|  |  |  |  |  |  |
|  |  | D276-K493 |  |  |  |
|  |  | K493-S275 | K493-S273 |  |  |
|  |  | K493-D276 |  |  |  |
| ***Inter-start (E-D and E-B)*** | | | | | |
| E642-K665 |  |  |  |  |  |
| K178-T175 |  |  | D181-K178 |  |  |
|  |  | N440-N442 |  |  |  |
|  |  | N440-N440 |  |  |  |
|  |  | N442-N442 |  |  |  |
|  |  | T443-T443 |  |  |  |
|  |  |  |  | T234-Y244 | T232-D244 |
|  |  |  |  |  | V235-D244 |
|  |  |  |  | Y244-G236 |  |
| **Protein-DNA** | | | | | |
| K685 | Y687 | K685 | Y687 |  |  |
| K344 | K342 | K344 | K342 |  |  |
| R324 | R322 | R324 | R322 |  |  |
| D82 | N80 | D82 | N80 |  |  |
| E321 | K319 | E321 | K319 |  |  |
|  | R322 |  | R322 |  |  |
| H343 |  | H343 |  |  |  |
