# Supplementary material for: The giant mimivirus 1.2 Mb genome is elegantly organized into a 30-nm diameter helical protein shield
Source: eLife. 2022 Jul 28;11:e77607. doi: 10.7554/eLife.77607 (PMC9512402; doi:10.7554/eLife.77607)
Supplement: Supplementary file 4. [file elife-77607-supp4.docx]

**Supplementary file 4: Data acquisition parameters for Cryo-EM**

|  | **Single-particle analysis** | **Tomograms** | **Bubblegrams** |
| --- | --- | --- | --- |
| **Hardware** | | |  |
| Microscope | Titan Krios | Titan Krios | Titan Krios |
| Detector | K2 | K3 | K3 |
| Accelerating voltage (kV) | 300 | 300 | 300 |
| Pixel size (Å) | 1.09 | 1.40 | 1.09 |
| **Data acquisition parameters** | | |  |
| Nominal magnification | 130,000 | 64,000 | 81,000 |
| Square pixel (Å²) | 1.1881 | 3.24 | 1.1881 |
| Dose per physical pixel per second | 7.5 | 15 | 15 |
| Flux (e-/Å²/s) | 6.3 | 4.6 | 12.64 |
| Exposure time (s) | 8 | 0.8 | 2-6 |
| Total exposure (e-/Å²) | 50.5 | 3.68 | 25-75 |
| Number of frames | 40 | 4 | 20-60 |
| Dose per frame (e-/Å²) | 1.25 | 0.92 | 1.25 |
| Defocus range (µm) | -1 to -3 | -2 to -4 | -3 to -5 |
| **Apertures (Size in microns)** | | |  |
| C1 | 2,000 | 2,000 | 2,000 |
| C2 | 70 | 50 | 50 |
| Microprobe/Nanoprobe | Np | Np | Np |
| Objective aperture size (µm) | 100 | 100 | 100 |
| **Energy filter** | | |  |
| Slit (eV) | 20 | 20 | 20 |
